# New diagnostic criteria and severity assessment of acute cholecystitis in revised Tokyo guidelines

**DOI:** 10.1007/s00534-012-0548-0

**Published:** 2012-08-08

**Authors:** Masamichi Yokoe, Tadahiro Takada, Steven M. Strasberg, Joseph S. Solomkin, Toshihiko Mayumi, Harumi Gomi, Henry A. Pitt, Dirk J. Gouma, O. James Garden, Markus W. Büchler, Seiki Kiriyama, Yasutoshi Kimura, Toshio Tsuyuguchi, Takao Itoi, Masahiro Yoshida, Fumihiko Miura, Yuichi Yamashita, Kohji Okamoto, Toshifumi Gabata, Jiro Hata, Ryota Higuchi, John A. Windsor, Philippus C. Bornman, Sheung-Tat Fan, Harijt Singh, Eduardo de Santibanes, Shinya Kusachi, Atsuhiko Murata, Xiao-Ping Chen, Palepu Jagannath, SungGyu Lee, Robert Padbury, Miin-Fu Chen

**Affiliations:** 1 General Internal Medicine, Nagoya Daini Red Cross Hospital, 2-9 Myoken-cho, Showa-ku, Nagoya, Aichi 466-8650 Japan; 2 Department of Surgery, Teikyo University School of Medicine, Tokyo, Japan; 3 Section of Hepatobiliary and Pancreatic Surgery, Washington University in Saint Louis School of Medicine, Saint Louis, MO USA; 4 Department of Surgery, University of Cincinnati College of Medicine, Cincinnati, OH USA; 5 Department of Emergency and Critical Care Medicine, Ichinomiya Municipal Hospital, Ichinomiya, Japan; 6 Center for Clinical Infectious Diseases, Jichi Medical University, Tochigi, Japan; 7 Department of Surgery, Indiana University School of Medicine, Indianapolis, IN USA; 8 Department of Surgery, Academic Medical Center, Amsterdam, The Netherlands; 9 Clinical Surgery, The University of Edinburgh, Edinburgh, UK; 10 Department of Surgery, University of Heidelberg, Heidelberg, Germany; 11 Department of Gastroenterology, Ogaki Municipal Hospital, Ogaki, Japan; 12 Department of Surgical Oncology and Gastroenterological Surgery, Sapporo Medical University School of Medicine, Sapporo, Japan; 13 Department of Medicine and Clinical Oncology, Graduate School of Medicine Chiba University, Chiba, Japan; 14 Department of Gastroenterology and Hepatology, Tokyo Medical University, Tokyo, Japan; 15 Clinical Research Center Kaken Hospital, International University of Health and Welfare, Ichikawa, Japan; 16 Department of Gastroenterological Surgery, Fukuoka University School of Medicine, Fukuoka, Japan; 17 Department of Surgery, Kitakyushu Municipal Yahata Hospital, Kitakyushu, Japan; 18 Department of Radiology, Kanazawa University Graduate School of Medical Science, Kanazawa, Japan; 19 Department of Endoscopy and Ultrasound, Kawasaki Medical School, Okayama, Japan; 20 Department of Surgery, Institute of Gastroenterology, Tokyo Women’s Medical University, Tokyo, Japan; 21 Department of Surgery, The University of Auckland, Auckland, New Zealand; 22 Division of General Surgery, Health Sciences, University of Cape Town, Cape Town, South Africa; 23 Department of Surgery, Queen Mary Hospital, The University of Hong Kong, Hong Kong, China; 24 Department of Hepato-Pancreato-Biliary Surgery, Hospital Selayang, Kuala Lampur, Malaysia; 25 Department of Surgery, Hospital Italianio, University of Buenos Aires, Buenos Aires, Argentina; 26 Department of Surgery, Toho University Medical Center Ohashi Hospital, Tokyo, Japan; 27 Department of Preventive Medicine and Community Health, School of Medicine, University of Occupational and Environmental Health, Kitakyushu, Japan; 28 Hepatic Surgery Centre, Department of Surgery, Tongji Hospital, Tongi Medical College, Huazhong University of Science and Technology, Wuhan, China; 29 Department of Surgical Oncology, Lilavati Hospital and Research Centre, Mumbai, India; 30 HepatoBiliary Surgery and Liver Transplantation, Asan Medical Center, Ulsan University, Ulsan, Korea; 31 Division of Surgical and Specialty Services, Flinders Medical Centre, Adelaide, Australia; 32 Chang Gung Memorial Hospital, Chang Gung University, Taoyuan, Taiwan

**Keywords:** Acute cholecystitis, Murphy’s sign, Diagnostic criteria, Severity assessment, Guidelines

## Abstract

**Background:**

The Tokyo Guidelines for the management of acute cholangitis and cholecystitis (TG07) were published in 2007 as the world’s first guidelines for acute cholangitis and cholecystitis. The diagnostic criteria and severity assessment of acute cholecystitis have since been widely used all over the world. A validation study of TG07 has shown that the diagnostic criteria for acute cholecystitis are highly reliable but that the definition of definite diagnosis is ambiguous. In addition, considerable new evidence referring to acute cholecystitis as well as evaluations of TG07 have been published. Consequently, we organized the Tokyo Guidelines Revision Committee to evaluate TG07, recognize new evidence, and conduct a multi-center analysis to revise the guidelines (TG13).

**Methods and materials:**

We retrospectively analyzed 451 patients with acute cholecystitis from multiple tertiary care centers in Japan. All 451 patients were first evaluated using the criteria in TG07. The “gold standard” for acute cholecystitis in this study was a diagnosis by pathology. The validity of TG07 diagnostic criteria was investigated by comparing clinical with pathological diagnosis.

**Results:**

Of 451 patients evaluated, a total of 227 patients were given a diagnosis of acute cholecystitis by pathological examination (prevalence 50.3 %). TG07 criteria provided a definite diagnosis of acute cholecystitis in 224 patients. The sensitivity of TG07 diagnostic criteria for acute cholecystitis was 92.1 %, and the specificity was 93.3 %. Based on the preliminary results, new diagnostic criteria for acute cholecystitis were proposed. Using the new criteria, the sensitivity of definite diagnosis was 91.2 %, and the specificity was 96.9 %. The accuracy rate was improved from 92.7 to 94.0 %. In regard to severity grading among 227 patients, 111 patients were classified as Mild (Grade I), 104 as Moderate (Grade II), and 12 as Severe (Grade III).

**Conclusion:**

The proposed new diagnostic criteria achieved better performance than the diagnostic criteria in TG07. Therefore, the proposed criteria have been adopted as new diagnostic criteria for acute cholecystitis and are referred to as the 2013 Tokyo Guidelines (TG13). Regarding severity assessment, no new evidence was found to suggest that the criteria in TG07 needed major adjustment. As a result, TG07 severity assessment criteria have been adopted in TG13 with minor changes.

## Introduction

Acute cholecystitis is a very common complication of cholelithiasis, and as such is frequently encountered in surgical practice [1–4]. There were no diagnostic criteria or severity assessment criteria for this common disease until 2007. In 2006, we conducted a systematic review and sponsored an international consensus conference in Tokyo, Japan. This meeting resulted in the development of the Tokyo Guidelines for the management of acute cholangitis and cholecystitis (TG07). These guidelines were the world’s first guidelines to include diagnostic criteria and severity assessment of acute cholecystitis [5] (Tables 1, 2).

**Table 1:** TG07 diagnostic criteria for acute cholecystitis

A. Local signs of inflammation, etc.:
(1) Murphy’s sign, (2) RUQ mass/pain/tenderness
B. Systemic signs of inflammation, etc.:
(1) Fever, (2) elevated CRP, (3) abnormal WBC count
C. Imaging findings: imaging findings characteristic of acute cholecystitis
Definite diagnosis
(1) One item in A + one item in B are positive
(2) C confirms the diagnosis when acute cholecystitis is suspected clinically

Acute hepatitis, other acute abdominal diseases, and chronic cholecystitis should be excluded

*WBC* white blood cell, *RUQ* right upper quadrant, *CRP* C-reactive protein

**Table 2:** TG07 severity assessment criteria for acute cholecystitis

“Severe” (Grade III) acute cholecystitis is accompanied by dysfunctions in any one of the following organs/systems:	
1. Cardiovascular dysfunction	Hypotension requiring treatment with dopamine ≥5 μg/kg per min, or any dose of dobutamine
2. Neurological dysfunction	Decreased level of consciousness
3. Respiratory dysfunction	PaO_2_/FiO_2_ ratio <300
4. Renal dysfunction	Oliguria, creatinine >2.0 mg/dl
5. Hepatic dysfunction	PT-INR >1.5
6. Hematological dysfunction	Platelet count <100,000/mm^3^
“Moderate” (Grade II) acute cholecystitis is accompanied by any one of the following conditions:	
1. Elevated WBC count (>18,000/mm^3^)	
2. Palpable tender mass in the right upper abdominal quadrant	
3. Duration of complaints >72 h^a^	
4. Marked local inflammation (gangrenous cholecystitis, pericholecystic abscess, hepatic abscess, biliary peritonitis, emphysematous cholecystitis)	
“Mild” (Grade I) acute cholecystitis does not meet the criteria of “Severe (Grade III)” or “Moderate (Grade II)” acute cholecystitis. Grade I can also be defined as acute cholecystitis in a healthy patient with no organ dysfunction and only mild inflammatory changes in the gallbladder, making cholecystectomy a safe and low-risk operative procedure.	

^a^Laparoscopic surgery in acute cholecystitis should be performed within 96 h after the onset

*WBC* white blood cell

Diagnostic and severity assessment criteria need to be updated periodically based on new information, criticisms, and suggestions for improvement. The diagnostic criteria in TG07 have been set to achieve high sensitivity in order to provide medical care suitable for a large number of patients. The sensitivity of TG07 diagnostic criteria has been reported to be 84.9 % [6] and TG07 diagnostic criteria are recognized as those to be recommended in current care for acute cholecystitis [1]. However, since its publication, we and others have found potential shortcomings in TG07 in clinical practice [6].

To update the Tokyo Guidelines for the management of acute cholangitis and cholecystitis, we organized the Tokyo Guidelines Revision Committee to evaluate TG07, recognize new evidence, and conduct a multi-center analysis to revise the guidelines (TG13). In the present study, we investigated the validity of the TG07 diagnostic criteria and severity assessment criteria by multi-center analysis. The limitations of TG07 were also investigated to develop tentative new diagnostic criteria and severity assessment criteria.

## Methods and materials

We retrospectively analyzed 451 patients from six tertiary care centers in Japan between November 2005 and November 2011: Sapporo Medical University, Tokyo Medical University, Tokyo Women’s Medical University, Nagoya Daini Red Cross Hospital, Ogaki Municipal Hospital, and Fukuoka University School of Medicine. Consecutive patients who were operated on for cholecystectomy were included during the study period. The “gold standard” for acute cholecystitis in this study was pathological diagnosis using standard gross and histological criteria. We therefore confirmed the final diagnosis by pathological examination of excised gallbladders after operation. If the pathological findings were chronic cholecystitis or other, those cases were considered to be “negative.”

All 451 patients were evaluated using TG07 criteria. The validity of the diagnostic criteria of TG07 was investigated by analyzing their sensitivity and specificity. The severity grading system of TG07 was evaluated by determining the distribution of severity among these patients. Through these data, the Tokyo Guidelines Revision Committee members discussed the quality of diagnostic criteria and severity assessment of acute cholecystitis in TG07 to reassess TG and propose new guidelines.

The literature was selected as follows: using “Tokyo Guidelines” AND “acute cholecystitis[MeSH]”, only 3 items were selected in PubMed since the publication of TG07 (1 April 2007 – 31 March 2012). These articles were screened with “human” and “English”. However, using “acute cholecystitis[MeSH]” AND “prognosis[MeSH]”, a total of 119 items were selected in PubMed over the same length of time. From these articles, the prognostic factors of acute cholecystitis to be utilized for the revision of TG07 were screened by the Tokyo Guidelines Revision Committee members. In addition, the distribution of severity grading was aggregated from the literature which reported the data based on the severity assessment of TG07.

The Tokyo Guidelines Revision Committee discussed the modification of TG07 diagnostic criteria and severity assessment of acute cholecystitis by analyzing the results of the present study and integrating the literature evidence.

## Results

### Assessment of TG07 diagnostic criteria and severity assessment criteria for acute cholecystitis

The 451 patients who were operated on for cholecystectomy comprised 255 male patients and 196 female patients with a mean age of 63.9 ± 14.0 years. 227 of the 451 patients enrolled were given a final pathological diagnosis of acute cholecystitis. The prevalence of acute cholecystitis in the cohort was 50.3 %. Based on the diagnostic criteria in TG07, a definite diagnosis of acute cholecystitis was made in 224 patients. Diagnostic criteria were not met in the remaining 227 patients.

We constructed 2 × 2 contingency tables between pathologically proven acute cholecystitis and acute cholecystitis diagnosed by TG07 criteria. There were 209 true-positive cases, 15 false-positive cases, 18 false-negative cases, and 209 true-negative cases (Table 3). The diagnostic sensitivity and specificity of TG07 were 92.1 % (209/227) and 93.3 % (209/224), respectively; the false-negative and false-positive rates were 7.9 % (18/227) and 6.7 % (15/224), respectively. The positive and negative predictive values were 93.3 and 92.1 %, respectively, and the positive and negative likelihood ratios were 13.75 and 0.08, respectively. The diagnostic accuracy was 92.7 %.

**Table 3:** 2 × 2 contingency tables of multi-center analysis for diagnostic criteria of TG07

		Acute cholecystitis by pathology		Total
		Yes	No	
TG07 definite diagnosis	Yes	209	15	224
	No	18	209	227
Total		227	224	451

In terms of severity assessment, a total of 227 patients who were given a final pathological diagnosis of acute cholecystitis were retrospectively examined with TG07 severity assessment criteria for acute cholecystitis. Of them, 111 patients were classified as Grade I (48.9 %), 104 patients as Grade II (45.8 %), and 12 patients as Grade III (5.3 %), respectively.

### Revision of TG07 diagnostic criteria and severity assessment criteria for acute cholecystitis

Several problems regarding the TG07 diagnostic criteria and severity assessment system were found during the analysis. The most important problem in TG07 was that the criteria for definite diagnosis were ambiguous and difficult to use. In TG07, there were two categories determining the definite diagnosis of acute cholecystitis (Table 1). Definite diagnosis 1: To obtain a definite diagnosis one item in A and one item in B had to be positive. Definite diagnosis 2: Imaging findings (Criterion C) confirmed the diagnosis when acute cholecystitis was suspected clinically. The Tokyo Guidelines Revision Committee concluded that the term “definite diagnosis” could not be supported in current practice without positive diagnostic imaging studies.

**Fig. 1:**
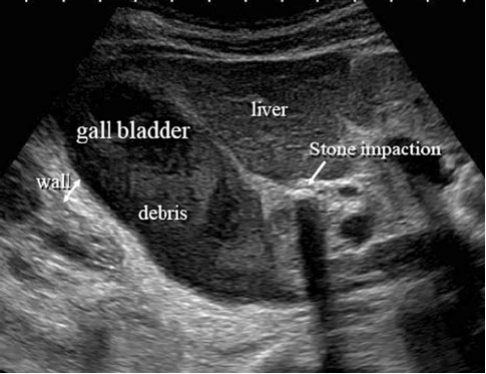
Typical US image of acute cholecystitis, demonstrating gallbladder swelling, wall thickening with sonolucent layers, massive debris, and the stone impaction in the cystic duct

For acute cholecystitis, abdominal ultrasonography, computed tomography (CT), and hepatobiliary scintigraphy (HIDA scan) are the imaging studies most commonly used in diagnosis. In particular, ultrasonography is a test that should be performed first of all in every case for which acute cholecystectomy is suspected. Sonography should be the screening test of choice in acute cholecystitis because it is cost-effective, prospectively highly accurate and fast [7]. The sensitivity of ultrasonography in detecting acute inflammation of the gallbladder has been reported to be 90–95 % [8]. Therefore, emergency room clinicians and surgeons currently prefer ultrasonography for the initial evaluation of suspected acute cholecystitis, because it is a simple, safe, fast and cost-effective tool [9–11]. Acute calculous cholecystitis is diagnosed radiologically by the concomitant presence of thickening of the gallbladder wall (5 mm or greater), pericholecystic fluid, or direct tenderness when the probe is pushed against the gallbladder (ultrasonographic Murphy’s sign) [1] (Fig. 1). CT findings of acute cholecystitis were reported as gallbladder distention (41 %), gallbladder wall thickening (59 %), pericholecystic fat density (52 %), pericholecystic fluid collection (31 %), subserosal edema (31 %) and high-attenuation gallbladder bile (24 %) [12].

As a result, the importance of diagnostic imaging was emphasized for the diagnosis of acute cholecystitis by the Committee, and new criteria were proposed (Table 4). In the proposed criteria a “suspected” diagnosis is achieved when one item from section A and one item from section B are present. A “definite” diagnosis is achieved when imaging findings characteristic of acute cholecystitis (Item C) are also present.

**Table 4:** TG13 diagnostic criteria for acute cholecystitis

A. Local signs of inflammation, etc.:
(1) Murphy’s sign, (2) RUQ mass/pain/tenderness
B. Systemic signs of inflammation, etc.:
(1) Fever, (2) elevated CRP, (3) elevated WBC count
C. Imaging findings:
Imaging findings characteristic of acute cholecystitis
Suspected diagnosis: One item in A + one item in B
Definite diagnosis: One item in A + one item in B + C

Acute hepatitis, other acute abdominal diseases, and chronic cholecystitis should be excluded

*RUQ* right upper quadrant, *CRP* C-reactive protein, *WBC* white blood cell

Regarding severity assessment criteria, a thorough literature search was performed and variables reported in the literature as predictive of poor prognosis in acute cholecystitis were summarized [13–25] (Table 5). The Tokyo Guidelines Revision Committee discussed whether these newly reported severity or prognostic factors such as diabetes mellitus, old age, and male sex should be adopted for revision. However, the Committee concluded that these factors were not supported by sufficient levels of evidence and so the factors were not adopted as assessment criteria. However, minor changes were made to the description of Grade III severity, i.e. dopamine and norepinephrine were both considered as evidence of cardiovascular dysfunction consistent with the SOFA score system [26] (Table 6).

**Table 5:** Prognostic factors in acute cholecystitis

Prognostic factor	Positive value	References
Leukocytosis		[13–19]
	≥15,000/mm^3^	[20]
	>14,900/mm^3^	[21]
	>13,000/mm^3^	[22]
	15,885/mm^3^ vs. 9,948/mm^3^	[23]
ALP		[17, 24, 25]
Age	>26 years old	[19]
	>45 years old	22
	>60 years old	[23]
Diabetes mellitus		[17, 20, 21]
Male		[20, 21]
Heart rate	>90 bpm	[22]
Gallbladder wall thickness >4.5 mm		[22]
Pericholecystic fluids		[17]
CBD dilatation		[25]
Admission delay		[18]

*ALP* alkaline phophatase, *CBD* common bile duct

**Table 6:** TG13 severity assessment criteria for acute cholecystitis

“Grade III” (severe) acute cholecystitis is associated with dysfunction of any one of the following organs/systems	
1. Cardiovascular dysfunction	Hypotension requiring treatment with dopamine ≥5 μg/kg per min, or any dose of norepinephrine
2. Neurological dysfunction	Decreased level of consciousness
3. Respiratory dysfunction	PaO_2_/FiO_2_ ratio <300
4. Renal dysfunction	Oliguria, creatinine >2.0 mg/dl
5. Hepatic dysfunction	PT-INR >1.5
6. Hematological dysfunction	Platelet count <100,000/mm^3^
“Grade II” (moderate) acute cholecystitis is associated with any one of the following conditions	
1. Elevated WBC count (>18,000/mm^3^)	
2. Palpable tender mass in the right upper abdominal quadrant	
3. Duration of complaints >72 h	
4. Marked local inflammation (gangrenous cholecystitis, pericholecystic abscess, hepatic abscess, biliary peritonitis, emphysematous cholecystitis)	
“Grade I” (mild) acute cholecystitis does not meet the criteria of “Grade III” or “Grade II” acute cholecystitis. Grade I can also be defined as acute cholecystitis in a healthy patient with no organ dysfunction and mild inflammatory changes in the gallbladder, making cholecystectomy a safe and low-risk operative procedure	

*WBC* white blood cell

### Assessment of TG13 diagnostic criteria and severity assessment for acute cholecystitis

Of 227 patients with a definite diagnosis of acute cholecystitis based on the proposed new diagnostic criteria, a final diagnosis of acute cholecystitis by pathology was made in 207 patients. We constructed 2 × 2 contingency tables between patients with acute cholecystitis by pathology and the cases with definite diagnosis using the proposed new diagnostic criteria, with 207 true-positive cases, 7 false-positive cases, 20 false-negative cases, and 217 true-negative cases (Table 7).

**Table 7:** 2 × 2 contingency tables of multi-center analysis for definite diagnosis of TG13 criteria

		Acute cholecystitis by pathology		Total
		Yes	No	
TG13 definite diagnosis	Yes	207	7	214
	No	20	217	237
Total		227	224	451

The diagnostic sensitivity and specificity of definite diagnosis were 91.2 % (207/227) and 96.9 % (217/224), respectively. The false-negative and false-positive rates were 8.8 % (20/227) and 3.1 % (7/224), respectively. The positive and negative predictive values were 96.7 and 91.6 %, respectively. The positive and negative likelihood ratios were 29.18 and 0.09, respectively. The diagnostic accuracy was 94.0 %. On the other hand, of the 219 patients with a suspected or definite diagnosis of acute cholecystitis based on the proposed new diagnostic criteria, a final diagnosis of acute cholecystitis was made in 208 patients. We constructed 2 × 2 contingency tables between patients with acute cholecystitis by pathology and the cases with suspected or definite diagnosis using the proposed new diagnostic criteria, with 208 true-positive cases, 11 false-positive cases, 19 false-negative cases, and 213 true-negative cases (Table 8). The diagnostic sensitivity and specificity of suspected or definite diagnosis were 91.6 % (208/227) and 95.1 % (213/224), respectively. The false-negative and false-positive rates were 8.4 % (19/227) and 4.9 % (11/224), respectively. The positive and negative predictive values were 95.0 and 91.8 %, respectively. The positive and negative likelihood ratios were 18.66 and 0.09, respectively. The diagnostic accuracy was 93.3 %.

**Table 8:** 2 × 2 contingency tables of multi-center analysis for a suspected or definite diagnosis of TG13 criteria

		Acute cholecystitis by pathology		Total
		Yes	No	
TG13 suspected or definite diagnosis	Yes	208	11	219
	No	19	213	232
Total		227	224	451

From those results the diagnostic validities were compared between the definite diagnosis of TG07 and that of the proposed new diagnostic criteria (Table 9).

**Table 9:** Comparison of TG07 and TG13 criteria for acute cholecystitis (*n* = 451, prevalence 50.3 %)

	TG07 (definite)	TG13 (definite)
Sensitivity (%)	92.1	91.2
Specificity (%)	93.3	96.9
False-negative (%)	7.9	8.8
False-positive (%)	6.7	3.1
Positive predictive value (%)	93.3	96.7
Negative predictive value (%)	92.1	91.6
Positive likelihood ratio	13.75	29.18
Negative likelihood ratio	0.08	0.09
Accuracy rate (%)	92.7	94.0

This comparison of the two diagnostic criteria in terms of diagnostic precision shows that the proposed new diagnostic criteria achieved better performance than TG07. These diagnostic criteria have therefore been chosen as the new diagnostic criteria of acute cholecystitis referred to as the Tokyo Guidelines (TG13).

On the other hand, the TG07 severity assessment criteria for acute cholecystitis did not have significant problems that required major revision of the definitions or structures. The TG07 severity assessment criteria have been adopted in the updated Tokyo Guidelines (TG13) with minor changes in descriptions as above (Table 6).

## Discussion

The most important role of diagnostic criteria and severity assessment is to allow early diagnosis and to provide the most appropriate treatment for the disease depending on its severity. TG07 of acute cholecystitis aimed at this by systematic literature search and integration of expert opinions through a consensus conference held in Tokyo in 2006 [27]. The guidelines should reflect the current clinical practice but they need periodic assessment and revision. However, in the case of TG07 this was particularly so because of shortcomings that became evident through application in clinical practice and as a result of new information in the literature.

For the diagnosis of acute cholecystitis, clinicians all over the world have provided treatment for acute cholecystitis based on Murphy’s sign. However, Murphy’s sign has been reported in previous studies to have a sensitivity of 50–60 % and a high specificity of 79 % [28] or 96 % [2] for the diagnosis of acute cholecystitis. The sensitivity of Murphy’s sign was once reported to be as low as 20.5 %, while the specificity was 87.5 % [6]. In the same study, the sensitivity and specificity of TG07 were as high as 84.9 and 50.0 %, respectively [6]. In this study, a sign test, which detects the difference in accuracy, was also performed to analyze statistically the diagnostic criteria of TG07 for acute cholecystitis and the rate of diagnostic accuracy of Murphy’s sign. The diagnostic accuracy was significantly higher when the TG07 were used than when Murphy’s sign was used [6]. TG07 can be used with more confidence among clinicians. However, the shortcomings of TG07 were recognized by the Tokyo Guidelines Revision Committee in that the definite diagnosis of TG07 had two categories. These two categories were ambiguous and the schema was difficult to use for many clinicians.

In addition to clinical and laboratory assessments, radiological and nuclear imaging techniques are widely used to identify individuals with complications of gallbladder disease [21]. Ultrasonographic diagnosis of acute cholecystitis was made when thickening and/or edema of gallbladder wall, distension of the gallbladder by gallstones, and pericholecystic fluid collection were seen [29].

Based on the above understanding, tentative new diagnostic criteria of acute cholecystitis were developed and their validity was analyzed among the patients from multiple institutions in Japan. Better diagnostic accuracy was obtained with the new criteria, with high sensitivity and high specificity on definite diagnosis. The new criteria validated by a retrospective analysis have been adopted as the revised diagnostic criteria of TG13 for acute cholecystitis.

The severity assessment criteria were reconsidered by the Tokyo Guidelines Revision Committee with new information, evidence, and evaluations of TG07. Lee et al. [14] revealed that there was a significantly shorter mean length of hospital stay in the patient group for whom the Tokyo guidelines (TG07) were utilized compared with those without compliance with TG07. Asai et al. [30] suggested that more precise severity grades may need to be established, including age and C-reactive protein as additional parameters.

The distribution of severity grading varies as follows: 39.3–68.5 % of the cases were classified as Grade I, 25.5–59.5 % as Grade II, and 1.2–6 % as Grade III [14, 30]. The present study shows that 48.9 % of the cases were classified as Grade I, 45.8 % as Grade II, and 5.3 % as Grade III. The proportions in the present study were not different from the proportions in other TG07 studies (Table 10).

**Table 10:** Distribution of severity grading of acute cholecystitis with TG07 and TG13

Severity assessment	Asai et al. [30] (TG07)	Lee et al. [14] (TG07)	Present study (TG13)
Severe (Grade III)	2 (1.2 %)	14 (6.0 %)	12 (5.3 %)
Moderate (Grade II)	97 (59.5 %)	60 (25.5 %)	104 (45.8 %)
Mild (Grade I)	64 (39.3 %)	161 (68.5 %)	111 (48.9 %)
Total	163	235	227

In summary, TG13 presents new diagnostic and severity assessment criteria based on a large patient base and a reasonable “gold standard”. These criteria allow early diagnosis and severity assessment of the disease and should be clinically very useful in the management of acute cholecystitis.

## Conclusion

The updated Tokyo Guidelines (TG13) introduce a new standard for the diagnosis and severity assessment of acute cholecystitis. In the TG13 diagnostic criteria, a “suspected” diagnosis is achieved when one item from section A and one item from section B are present. A “definite” diagnosis is achieved when imaging findings characteristic of acute cholecystitis (Item C) are also present. Compared with TG07, the validity of the diagnostic criteria has been improved and the severity assessment criteria of TG07 have been adopted with minor changes from TG07.
